# Use of Menthol-Flavored Tobacco Products Among US Middle and High School Students: National Youth Tobacco Survey, 2022

**DOI:** 10.5888/pcd21.230305

**Published:** 2024-05-30

**Authors:** Monica E. Cornelius, Andrea S. Gentzke, Caitlin G. Loretan, Nikki A. Hawkins, Ahmed Jamal

**Affiliations:** 1 Office on Smoking and Health, National Center for Chronic Disease Prevention and Health Promotion, Centers for Disease Control and Prevention, Atlanta, Georgia

## Abstract

**Introduction:**

Menthol cigarettes have been associated with increased smoking initiation. Although numerous studies have focused on correlates of menthol cigarette smoking among youths, fewer studies have assessed the prevalence and correlates of overall menthol-flavored tobacco product use among middle and high school students.

**Methods:**

We analyzed 2022 National Youth Tobacco Survey data to estimate the prevalence of menthol-flavored tobacco product use among US middle and high school students who used tobacco products within the past 30 days. Characteristics associated with menthol-flavored tobacco product use were also examined.

**Results:**

Use of menthol-flavored tobacco products was reported by 23.8% of students who currently used any tobacco product and by 39.5% of students who currently used any flavored tobacco product. Among students who reported past 30-day use of a flavored tobacco product, characteristics associated with a higher prevalence of menthol-flavored tobacco product use included non-Hispanic White race and ethnicity, frequent tobacco product use, use of multiple tobacco products, wanting to use a tobacco product within the first 30 minutes of awakening, and craving tobacco products within the past 30 days.

**Conclusion:**

Unlike results of prior research focused on cigarette smoking among young people, prevalence of use of any menthol-flavored tobacco product was highest among non-Hispanic White youths. Any use of menthol-flavored tobacco products of any type (alone or in combination with other flavors) among young people may be associated with continued product use and symptoms of dependence.

**Summary**

**What is already known on this topic?**

Middle and high school students who currently use tobacco products report using a variety of flavors, including menthol.

**What is added by this report?**

In 2022, 23.8% of students who currently used any tobacco product and 39.5% who currently used flavored tobacco products reported using menthol-flavored tobacco products. Students who exhibited characteristics of addiction to tobacco product use had a higher prevalence of menthol-flavored tobacco product use.

**What are the implications for public health practice?**

Menthol and other characterizing flavors or additives in tobacco products may contribute to first-time tobacco use and sustained use among young people. Understanding this association can inform public health policy aimed at preventing and reducing tobacco product use in this population.

## Introduction

Menthol, an additive in commercial tobacco products, creates a cooling sensation when inhaled (1–3). Menthol has both flavor and sensation properties (1–3). The effects of menthol can make cigarette smoke or e-cigarette aerosol seem less irritating and can enhance the product-user’s experience (1–4). Menthol flavoring is not limited to cigarettes and e-cigarettes; most types of commercial tobacco products are available in menthol flavor (3). Menthol cigarettes have been associated with increased smoking initiation, nicotine dependence, and lower smoking cessation success (1,3,5). Results from modeling studies suggest that prohibiting menthol cigarettes in the US could result in a 15% reduction in smoking prevalence and prevent an estimated 324,000 to 654,000 deaths over the next 40 years (6–8).

Disparities among population groups that use menthol cigarettes are well-documented. Marketing directed at certain population groups has been associated with a higher prevalence of menthol cigarette smoking in these groups (1,3,9,10). Population groups most likely to smoke menthol cigarettes are non-Hispanic Black people, women, sexual minority groups, people identifying as transgender, people residing in low-income communities, people with mental health conditions, youths, and young adults (3).

Smoking initiation usually begins in adolescence (4) when use of nicotine can have negative consequences on brain development and may increase the risk for nicotine dependence (11). Middle and high school students often use a variety of commercial tobacco products available in flavors, including menthol (12). E-cigarettes are the most commonly used tobacco product among middle and high school students — with 9.4% reporting e-cigarette use in 2022 — followed by cigars (1.9%) and cigarettes (1.6%) (12,13). Almost 4 of 5 (79.1%) middle and high school students who reported current use of 1 or more tobacco products used a flavored tobacco product (12). Furthermore, among middle and high school students who currently used any flavored tobacco product, 38.8% reported smoking menthol cigarettes (12). Non-Hispanic Black, Hispanic, and female middle and high school students have reported a higher prevalence of smoking menthol cigarettes (14).

Although numerous studies have focused on correlates of menthol cigarette smoking among youths, fewer studies have assessed the prevalence of using both cigarette and noncigarette menthol-flavored tobacco products in this population (14,15). Such information is important because, although the prevalence of cigarette smoking among youths has declined, use of e-cigarettes has increased, and new tobacco product types (eg, heated tobacco products) continue to become available (13,14). To examine whether previously observed characteristics associated with menthol cigarette smoking (eg, higher prevalence among Black, Hispanic, and female adolescent populations) are similar for use of any menthol-flavored tobacco product among adolescents, our study will 1) provide updated estimates of menthol-flavored tobacco product use among middle and high school students and 2) assess correlates of use of any menthol-flavored tobacco products in this population. Assessing correlates of menthol-flavored tobacco product use among youths can further identify populations that may benefit from public health strategies recognizing the effects of flavored tobacco products in reducing tobacco product use by young people.

## Methods

### Data sample

We analyzed data from the 2022 National Youth Tobacco Survey (NYTS), a cross-sectional, school-based, voluntary, self-administered survey of US middle and high school students in grades 6 to 12 (12,13). A stratified 3-stage cluster sampling procedure generated a nationally representative sample of US students attending public and private schools (16). We collected data from January through May 2022 from 28,291 middle and high school students (overall response rate: 45.2%) by using a web-based survey with 99.3% of respondents completing the survey on a school campus. The analytic sample consisted of middle and high school students who reported use of 1 or more tobacco products within the past 30 days. The 2022 NYTS was approved by the institutional review boards of the data collectors, the CDC institutional review board (45 C.F.R. part 46; 21 C.F.R. part 56), and the Office of Management and Budget.

### Measures

We assessed current use of menthol-flavored tobacco products among students who indicated past 30-day use of any tobacco product (use of ≥1 tobacco products: e-cigarettes, cigarettes, cigars, smokeless tobacco [chewing tobacco, snuff, dip, snus], dissolvable tobacco products, waterpipes or hookahs, pipe tobacco, bidis, heated tobacco products, or nicotine pouches). We also assessed use of menthol-flavored tobacco products among students who indicated past 30-day use of any flavored tobacco products. Menthol-flavored tobacco product use was defined as using any menthol-flavored tobacco product within the past 30 days, regardless of whether other flavors of tobacco products were used. Responses of “yes” to questions about flavored tobacco product use and “menthol” to the type(s) of flavor used were categorized as menthol-flavored tobacco use. For cigarettes, respondents who, within the past 30 days 1) indicated using only 1 cigarette brand and indicated that the brand was a menthol-flavored brand (Kool, Newport), 2) responded that they smoked Kool or Newport brands to the question “During the past 30 days, what brand of cigarettes did you usually smoke? (Choose only one answer)” (asked among respondents who used multiple brands in the past 30 days), or 3) who answered yes to “During the past 30 days, were the cigarettes that you usually smoked menthol?” were considered to have used menthol-flavored tobacco products (12). Students indicating no use of menthol-flavored tobacco products were categorized as using nonmenthol tobacco products.

Among students who used a flavored tobacco product in the past 30 days, tobacco product use was categorized as follows: 1) e-cigarettes only; 2) combustible tobacco products (cigarettes, cigars, bidis, hookahs, or pipes) only; 3) other tobacco products (smokeless tobacco products [chewing tobacco, snuff, dip, snus], dissolvables, heated tobacco products, or nicotine pouches) only; and 4) any combination of the preceding 3 categories.

Covariates examined included sex (male/female), race and ethnicity (Hispanic, non-Hispanic Black, non-Hispanic White, non-Hispanic Other), sexual orientation (heterosexual, lesbian, gay, bisexual, not sure), transgender identity (yes, no, not sure, don’t know what question is asking), family affluence (scores of low [0–5], medium [6,7], high [8,9] on a 4-item scale), tobacco product advertising exposure (yes [most of the time/always/sometimes], no [rarely/none]), frequent use (≥20 of the past 30 days) of a tobacco product, use of multiple tobacco products (≥2 products), time to wanting to use a tobacco product after awakening (<30 minutes, ≥30 minutes), craving tobacco products within the past 30 days (yes, no), past-year quit attempts, and quit intentions. Categorization of family affluence, advertising exposure, and cessation behaviors were consistent with previous analyses (12).

Respondents who indicated seeing advertisements or promotions for e-cigarettes, cigarettes, and other tobacco products “sometimes,” “most of the time,” or “always” on the internet, in newspapers or magazines, at a store (convenience store, supermarket, gas station, kiosk/storefront, or shopping center), or on television or streaming services were categorized as having been exposed to tobacco product advertising. Those who responded “never” or “rarely” were categorized as unexposed. Those who reported “I do not use the internet,” “I do not read newspapers or magazines,” “I never go to a convenience store, supermarket, or gas station,” or “I do not watch television or streaming services or go to the movies” were excluded.

Respondents who indicated 1 or more for the number of times they had stopped using all tobacco products for 1 day or longer because they were trying to quit were categorized as having a past-year quit attempt. Those who indicated “I did not try to quit all tobacco products during the past 12 months” were categorized as not having made a past-year quit attempt. Respondents who indicated they were seriously thinking about quitting the use of all tobacco products were categorized as having quit intentions; those that responded “No, I am not thinking about quitting the use of all tobacco products” were categorized as not having quit intentions.

### Analysis 

We computed the weighted prevalence and 95% CIs separately for menthol-flavored and nonmenthol-flavored tobacco product use among students who used 1) 1 or more tobacco products within the past 30 days (n = 3,334) and 2) 1 or more flavored tobacco products within the past 30 days (n = 2,020), overall and by sociodemographic characteristics, tobacco use characteristics, cessation behaviors, and advertising exposure. We also computed the weighted percentage of menthol use by type of tobacco product. Additionally, we computed the percentage of each characteristic by menthol and nonmenthol tobacco product use among students who used flavored tobacco products (n = 2,020), which is the primary focus of our study. Chi-square tests of independence were used to test for differences in the proportions of each characteristic among menthol- and nonmenthol-flavored tobacco product use, with a *P* value of <.05 indicating significance. Nested logistic regression models (unadjusted models and models adjusted for sex, racial or ethnic group, and grade level) were used to estimate associations between each characteristic of interest and current use of menthol-flavored tobacco products among students who used 1 or more flavored tobacco products within the past 30 days. Model-adjusted prevalence ratios (APRs) with predicted marginals and Wald χ^2^ statistics were computed. Models were adjusted to control for confounding in the associations between each covariate of interest and menthol-flavored tobacco product use. All analyses were performed using SAS-callable SUDAAN software, version 11.0.3 (RTI International).

## Results

### Prevalence of menthol-flavored and nonmenthol-flavored tobacco product use

**Nonmenthol- and menthol-flavored tobacco product use among students who used any tobacco products. **In 2022, 3.1 million middle and high school students (11.3%) reported currently using any tobacco product. Most of these students reported using nonmenthol tobacco products (76.2%), ranging from 56.0% (those indicating a time of wanting to use a tobacco product after awakening of <30 min) to 92.2% (non-Hispanic Black students) (Table 1). Among middle and high school students who reported current use of any tobacco product, 23.8% (an estimated 730,000 students) reported use of a menthol-flavored tobacco product; prevalence of menthol-flavored tobacco product use was 25.6% among males and 22.2% among females (Table 1). Prevalence of menthol-flavored tobacco product use by race or ethnicity ranged from 7.8% among non-Hispanic Black students to 30.1% among non-Hispanic White students. Prevalence was 19.6% among middle school students and 24.3% among high school students. Prevalence of menthol-flavored tobacco product use across sexual orientation categories ranged from 24.4% to 26.5%. Prevalence of menthol-flavored tobacco product use by transgender identity ranged from 20.5% among students who didn’t know what the question was asking to 37.7% among students who identified as transgender. Prevalence of menthol-flavored tobacco use among students with characteristics indicative of tobacco addiction (frequent use of tobacco, craving tobacco products, use of multiple tobacco products, and time after awakening to wanting to use a tobacco product) ranged from 38.0% to 44.0% compared with 13.8% to 23.5% among students who did not report characteristics indicative of tobacco addiction. Prevalence of menthol-flavored tobacco use was 26.5% among students with exposure to tobacco product advertising, 24.8% among students who intended to quit using all tobacco products, and 26.2% among students who reported a past-year quit attempt.

**Table 1:** Prevalence of Current Menthol- and Nonmenthol-Flavored Tobacco Product Use^a^ Among Middle and High School Students Who Use Tobacco Products, by Sociodemographic Characteristics and Cessation Behaviors, National Youth Tobacco Survey, United States, 2022

Characteristic	Estimated no. of students who used menthol-flavored product^b^	All students who currently use any tobacco product (n = 3,334)		All students who currently use any flavored tobacco product (n = 2,020)	
		Prevalence, use of any menthol-flavored products, % (95% CI)	Prevalence, use of only nonmenthol-flavored products, % (95% CI)	Prevalence, use of any menthol-flavored products, % (95% CI)	Prevalence, use of only nonmenthol-flavored products, % (95% CI)
**All students**	730,000	23.8 (19.7–28.5)	76.2 (71.5–80.3)	39.5 (34.0–45.3)	60.5 (54.7–66.0)
**Demographic characteristic**					
**Sex**					
Male	360,000	25.6 (20.7–31.2)	74.4 (68.8–79.3)	43.7 (37.2–50.5)	56.3 (49.5–62.8)
Female	360,000	22.2 (17.6–27.5)	77.8 (72.5–82.4)	35.9 (29.5–42.8)	64.1 (57.2–70.5)
**Race or ethnicity**					
Hispanic	130,000	16.6 (12.3–22.0)	83.4 (78.0–87.7)	28.5 (21.9–36.3)	71.5 (63.7–78.1)
Non-Hispanic Black	20,000	7.8 (4.7–12.6)	92.2 (87.4–95.3)	15.5 (9.5–24.2)	84.5 (75.8–90.5)
Non-Hispanic White	480,000	30.1 (25.4–35.3)	69.9 (64.7–74.6)	47.1 (40.7–53.6)	52.9 (46.4–59.3)
Non-Hispanic Other	70,000	26.0 (18.7–34.8)	74.0 (65.2–81.3)	43.8 (33.1–55.1)	56.2 (44.9–66.9)
**Grade level**					
Middle school (grades 6–8)	100,000	19.6 (14.6–25.9)	80.4 (74.1–85.4)	34.7 (27.3–42.9)	65.3 (57.1–72.7)
High school (grades 9–12)	610,000	24.3 (19.8–29.4)	75.7 (70.6–80.2)	39.9 (33.8–46.2)	60.1 (53.8–66.2)
**Sexual orientation**					
Heterosexual	420,000	24.4 (19.7–29.8)	75.6 (70.2–80.3)	39.4 (32.9–46.4)	60.6 (53.6–67.1)
Gay, lesbian, or bisexual	160,000	26.2 (20.4–33.0)	73.8 (67.0–79.6)	39.6 (31.6–48.3)	60.4 (51.7–68.4)
Not sure	40,000	26.5 (19.3–35.3)	73.5 (64.7–80.7)	44.3 (30.6–59.0)	55.7 (41.0–69.4)
**Transgender identity**					
No, not transgender	520,000	23.6 (19.5–28.4)	76.4 (71.6–80.5)	37.6 (32.0–43.5)	62.4 (56.5–68.0)
Yes, transgender	50,000	37.7 (26.3–50.5)	62.3 (49.5–73.7)	56.3 (38.5–72.6)	43.7 (27.5–61.5)
Not sure	40,000	36.6 (24.1–51.3)	63.4 (48.7–75.9)	58.8 (39.1–76.1)	41.2 (23.9–60.9)
I don’t know what this question is asking	10,000	20.5 (11.5–33.9)	79.5 (66.1–88.5)	38.3 (22.4–57.2)	61.7 (42.8–77.6)
**Family affluence scale^c^**					
Low	150,000	22.4 (16.8–29.4)	77.6 (70.6–83.2)	39.6 (30.5–49.4)	60.4 (50.6–69.5)
Medium	190,000	22.0 (17.4–27.3)	78.0 (72.7–82.6)	36.7 (29.0–45.1)	63.3 (54.9–71.0)
High	250,000	28.3 (22.1–35.4)	71.7 (64.6–77.9)	41.3 (33.3–49.8)	58.7 (50.2–66.7)
**Tobacco product advertising exposure^d^**					
Yes (most of the time/always/sometimes)	590,000	26.5 (21.4–32.2)	73.5 (67.8–78.6)	41.2 (34.7–48.1)	58.8 (51.9–65.3)
No (rarely/never)	70,000	15.8 (10.5–23.1)	84.2 (76.9–89.5)	31.5 (21.4–43.7)	68.5 (56.3–78.6)
**Frequent tobacco product use^e^**					
Yes	480,000	38.0 (31.7–44.7)	62.0 (55.3–68.3)	53.1 (45.9–60.1)	46.9 (39.9–54.1)
No	240,000	13.8 (11.1–17.1)	86.2 (82.9–88.9)	26.4 (21.8–31.6)	73.6 (68.4–78.2)
**Use multiple tobacco products**					
Yes	390,000	41.1 (35.4–47.2)	58.9 (52.8–64.6)	53.0 (46.7–59.2)	47.0 (40.8–53.3)
No	330,000	15.9 (12.1–20.7)	84.1 (79.3–87.9)	30.4 (23.8–37.9)	69.6 (62.1–76.2)
**Time to wanting to use a tobacco product <30 min after awakening**					
Yes	250,000	44.0 (34.5–53.8)	56.0 (46.2–65.5)	57.9 (46.9–68.2)	42.1 (31.8–53.1)
No	270,000	23.5 (19.2–28.4)	76.5 (71.6–80.8)	36.5 (30.4–43.0)	63.5 (57.0–69.6)
**Craving tobacco products within the past 30 days^f^**					
Yes	310,000	38.9 (31.2–47.2)	61.1 (52.8–68.8)	50.7 (42.0–59.5)	49.3 (40.5–58.0)
No	380,000	18.7 (15.1–22.9)	81.3 (77.1–84.9)	33.4 (27.9–39.3)	66.6 (60.7–72.1)
**Past-year quit attempt^g^**					
Yes	400,000	26.2 (20.9–32.3)	73.8 (67.7–79.1)	40.6 (34.0–47.6)	59.4 (52.4–66.0)
No	260,000	25.9 (20.5–32.0)	74.1 (68.0–79.5)	41.6 (33.8–49.8)	58.4 (50.2–66.2)
**Quit intentions^h^**					
Yes	400,000	24.8 (19.8–30.7)	75.2 (69.3–80.2)	38.3 (31.4–45.6)	61.7 (54.4–68.6)
No	260,000	27.5 (23.1–32.2)	72.5 (67.8–76.9)	45.4 (39.5–51.4)	54.6 (48.6–60.5)

a Current menthol-flavored tobacco product use was assessed among students who indicated past 30-day tobacco product use (use of ≥1 tobacco products including e-cigarettes, cigarettes, cigars, smokeless tobacco [chewing tobacco, snuff, dip, snus], dissolvable tobacco products, waterpipes/hookahs, pipe tobacco, bidis, heated tobacco products, and nicotine pouches). Those responding “yes” to using a flavored product and “menthol” to type of flavor were categorized as having used menthol-flavored tobacco products. For cigarettes, respondents who, within the past 30 days, indicated 1) using only 1 cigarette brand and indicated that the brand was a menthol-flavored brand (Kool, Newport); 2) responded that they smoked Kool or Newport brands to the question “During the past 30 days, what brand of cigarettes did you usually smoke? (Choose only 1 answer)” (asked among respondents who used multiple brands in the past 30 days); or 3) who answered “yes” to “During the past 30 days, were the cigarettes that you usually smoked menthol?” were considered as having using menthol-flavored tobacco products.

b Estimated weighted total numbers were rounded to the nearest 10,000 persons. Overall population estimates might not sum to corresponding subgroup population estimates because of rounding or inclusion of students who did not self-report sex, race and ethnicity, or grade level.

c Family affluence was assessed with a composite scale that comprised 4 questions: 1) “Does your family own a vehicle (such as a car, van, or truck)?”; 2) “Do you have your own bedroom?”; 3) “How many computers (including laptops and tablets, not including game consoles and smartphones) does your family own?”; and 4) “During the past 12 months, how many times did you travel on vacation with your family?” Complete data from all 4 questions (n=2,619 among students who currently use tobacco products; n = 1,617 among students who currently used flavored tobacco products) were summed (range, 0–9) and categorized into approximate tertiles based on the sample’s weighted distribution of scores.

d Exposure to tobacco product marketing (advertisements or promotions) was assessed separately for e-cigarettes, cigarettes, and other tobacco products for 4 sources: retail stores; internet; television, streaming services, or movies; and newspapers or magazines. Respondents were asked, “When you [are using the Internet; read newspapers or magazines; go to a convenience store, supermarket, or gas station; watch television or streaming services (such as Netflix, Hulu, or Amazon Prime), or go to the movies], how often do you see ads or promotions for [e-cigarettes; cigarettes or other tobacco products]?” Respondents were categorized as exposed if they responded “sometimes,” “most of the time,” or “always” or unexposed if they responded “never” or “rarely.” Those who reported “I do not use the internet,” “I do not read newspapers or magazines,” “I never go to a convenience store, supermarket, or gas station,” or “I do not watch television or streaming services or go to the movies” were excluded from the analysis. There were 476 respondents excluded among students reporting current tobacco product use and 262 respondents excluded among students reporting current flavored tobacco product use.

e People who used tobacco products in the past 30 days who indicated use of any product on 20 or more days in the past 30 days were categorized as using tobacco products frequently; otherwise, if all tobacco products were reported as being used less than 20 days out of the last 30, they were categorized as not having frequent tobacco product use.

f Based on the question “During the past 30 days, have you had a strong craving or felt like you really needed to use a tobacco product of any kind?” Those answering “yes” were categorized as craving tobacco products within the past 30 days.

g Based on the question, “During the past 12 months, how many times have you stopped using all tobacco products for 1 day or longer because you were trying to quit tobacco products for good?” Responses other than “I did not try to quit all tobacco products during the past 12 months” were considered having made 1 or more quit attempts. Respondents missing data on this outcome (n = 619 among students reporting current tobacco product use; n = 286 among students reporting current flavored tobacco product use) were excluded from the analysis.

h Based on the question, “Are you seriously thinking about quitting the use of all tobacco products?” Responses of “Yes, during the next 30 days,” “Yes, during the next 6 months,” “Yes, during the next 12 months,” and “Yes, but not during the next 12 months” indicated having quit intentions. The response, “No, I am not thinking about quitting the use of all tobacco products” indicated not having quit intentions. Respondents missing data on this outcome (n = 578 among students reporting current tobacco product use; n = 265 among students reporting current flavored tobacco product use) were excluded from the analysis.

**Nonmenthol- and menthol-flavored tobacco product use among students who used flavored tobacco products. **Most students who currently used any flavored tobacco product reported using nonmenthol tobacco products (60.5%), ranging from 41.2% (those indicating “not sure” if they were transgender) to 84.5% (non-Hispanic Black students) (Table 1). Among students who reported current use of a flavored tobacco product, 39.5% reported use of menthol-flavored tobacco products (Table 1) (Figure). Among middle and high school students who currently used any flavored tobacco products, prevalence of menthol-flavored tobacco product use by sex was 43.7% among males and 35.9% among females (Table 1). Prevalence of menthol-flavored tobacco product use ranged from 15.5% among non-Hispanic Black students to 47.1% among non-Hispanic White students. Among middle school students, the prevalence was 34.7% compared with 39.9% among high school students and ranged from 39.4% to 44.3% across sexual orientation categories. Prevalence of menthol-flavored tobacco product use by transgender identity ranged from 37.6% among those who identified as not transgender to 58.8% among those who were not sure. Prevalence of menthol-flavored tobacco use among students with characteristics indicative of addiction (craving tobacco products, use of multiple tobacco products, frequent use of tobacco, and time after awakening to wanting to use a tobacco product) ranged from 50.7% to 57.9% compared with 26.4% to 36.5% among students who did not report characteristics indicative of tobacco addiction. Prevalence of menthol-flavored tobacco use was 41.2% among students with exposure to tobacco product advertising, 38.3% among students who intended to quit using all tobacco products, and 40.6% among students who reported a past-year quit attempt.

**Figure:**
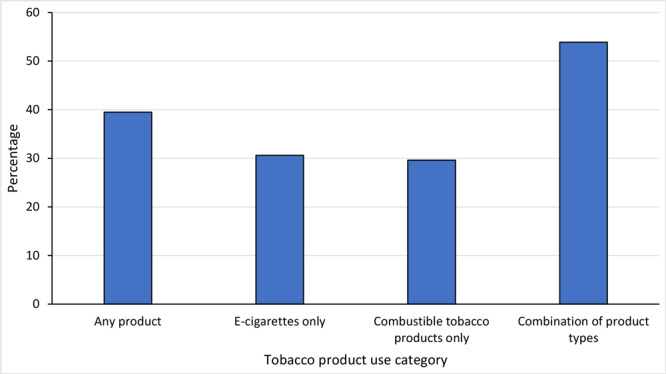
Use of menthol-flavored tobacco products, by current type of tobacco product used, among middle and high school students who currently used flavored tobacco products (N = 2,020), National Youth Tobacco Survey, United States, 2022.

**Table:** 

Type of menthol-flavored tobacco product	Menthol tobacco use, by product, %
Any product	39.5%
E-cigarettes only	30.6%
Combustible tobacco products only	29.6%
Combination of product types	53.9%

**Menthol-flavored tobacco use by type of flavored tobacco product. **Approximately 53.9% of students who used a combination of types of flavored tobacco products, including e-cigarettes, combustible tobacco products, and other types of tobacco product, indicated use of at least 1 menthol-flavored tobacco product (Figure). Among students who exclusively used e-cigarettes, 30.6% reported using menthol-flavored products, and 29.6% of students who exclusively used combustible tobacco products reported using menthol-flavored products. The estimate for prevalence of use of menthol-flavored tobacco products among students who exclusively used other types of tobacco products was not statistically reliable and is not presented.

**Characteristics of middle and high school students who use menthol- and nonmenthol-flavored tobacco products among students who use flavored tobacco products.** Among students who used any flavored tobacco products, those who used menthol-flavored products differed from those who used nonmenthol-flavored products (Table 2). Compared with students who used nonmenthol-flavored tobacco products, a higher proportion of students who used menthol-flavored tobacco products were male (50.4% among menthol vs 42.2% among nonmenthol, *P* = .04) or non-Hispanic White, Hispanic, or non-Hispanic Other (96.2% menthol vs 86.5% nonmenthol, *P* < .001, not shown in table). In contrast, compared with students who used nonmenthol-flavored products, a lower proportion of students who used menthol-flavored products were non-Hispanic Black (3.8% menthol vs 13.5% nonmenthol, *P* < .001). A higher proportion of students who used menthol-flavored tobacco products (compared with students who used nonmenthol-flavored products) used tobacco products frequently (66.0% vs 38.1%, *P* < .001); used multiple tobacco products (54.0% vs 31.3%, *P* < .001); wanted to use a tobacco product within less than 30 minutes of awakening (48.1% vs 27.9%, *P* < .001); craved tobacco products within the past 30 days (44.8% vs 28.3%, *P* < .001); and did not intend to quit using tobacco products (39.9% vs 33.1%, *P* = .03).

**Table 2:** Use of Menthol^a^ and Nonmenthol-Flavored Tobacco Products Among Middle and High School Students Who Currently Use Any Flavored Tobacco Products, by Selected Characteristics and Tobacco Use Behaviors, National Youth Tobacco Survey, 2022

Characteristic	Total estimated no. who currently use tobacco products^b^	Any menthol flavor		Only nonmenthol flavors		*P* value^c^
		Estimated no.^b^	% (95% CI)	Estimated no.^b^	% (95% CI)	
**All students**	1,850,000	730,000	39.5 (34.0–45.3)	1,120,000	60.5 (54.7–66.0)	Not applicable
**Demographic characteristic**						
**Overall**						
**Sex**						
Male	830,000	360,000	50.4 (42.6–58.2)	470,000	42.2 (37.4–47.2)	.04
Female	1,000,000	360,000	49.6 (41.8–57.4)	640,000	57.8 (52.8–62.6)	
**Race or ethnicity**						
Hispanic	460,000	130,000	18.4 (14.0–23.8)	330,000	29.5 (23.4–36.5)	<.001
Non-Hispanic Black	170,000	20,000	3.8 (2.5–5.8)	150,000	13.5 (9.4–18.9)	
Non-Hispanic White	1,020,000	480,000	67.3 (60.8–73.3)	540,000	48.5 (39.7–57.3)	
Non-Hispanic Other	170,000	70,000	10.4 (7.1–15.1)	90,000	8.5 (6.1–11.9)	
**Grade level**						
Middle school (grades 6–8)	300,000	100,000	14.8 (10.0–21.2)	190,000	17.8 (12.7–24.4)	.23
High school (grades 9–12)	1,530,000	610,000	85.2 (78.8–90.0)	920,000	82.2 (75.6–87.3)	
**Sexual orientation**						
Heterosexual	1,080,000	420,000	66.8 (61.1–72.0)	650,000	67.8 (62.2–72.9)	.79
Gay, lesbian, or bisexual	420,000	160,000	26.1 (21.2–31.7)	250,000	26.3 (21.5–31.8)	
Not Sure	100,000	40,000	7.1 (4.9–10.3)	50,000	5.9 (3.8–9.0)	
**Transgender identity**						
No, not transgender	1,380,000	520,000	82.0 (75.9–86.8)	860,000	89.6 (86.0–92.4)	.11
Yes, transgender	90,000	50,000	8.2 (5.2–12.7)	40,000	^—d^	
Not sure	70,000	40,000	7.1 (4.6–10.7)	30,000	3.3 (1.9–5.5)	
I don’t know what this question is asking	40,000	10,000	—d	20,000	—d	
**Family affluence scale^e^**						
Low	380,000	150,000	25.6 (19.0–33.5)	230,000	25.3 (20.7–30.5)	.73
Medium	520,000	190,000	32.0 (25.8–39.0)	330,000	35.8 (29.9–42.1)	
High	610,000	250,000	42.4 (34.8–50.3)	360,000	38.9 (31.9–46.4)	
**Tobacco product advertising exposure^f^**						
Yes (most of the time/always/sometimes)	1,440,000	590,000	89.3 (83.8–93.1)	840,000	84.6 (79.9–88.3)	.20
No (rarely/never)	220,000	70,000	10.7 (6.9–16.2)	150,000	15.4 (11.7–20.1)	
**Frequent tobacco product use^g^**						
Yes	910,000	480,000	66.0 (61.4–70.4)	420,000	38.1 (33.7–42.7)	<.001
No	940,000	240,000	34.0 (29.6–38.6)	690,000	61.9 (57.3–66.3)	
**Use of multiple tobacco products**						
Yes	740,000	390,000	54.0 (47.6–60.4)	350,000	31.3 (26.4–36.7)	<.001
No	1,100,000	330,000	46.0 (39.6–52.4)	770,000	68.7 (63.3–73.6)	
**Time to wanting to use a tobacco product <30 min after awakening**						
Yes	440,000	250,000	48.1 (41.3–55.0)	180,000	27.9 (23.3–33.0)	<.001
No	760,000	270,000	51.9 (45.0–58.7)	480,000	72.1 (67.0–76.7)	
**Craving tobacco products within the past 30 days^h^**						
Yes	610,000	310,000	44.8 (36.8–53.0)	300,000	28.3 (24.5–32.3)	<.001
No	1,150,000	380,000	55.2 (47.0–63.2)	760,000	71.7 (67.7–75.5)	
**Past-year quit attempt^i^**						
Yes	990,000	400,000	60.3 (53.8–66.6)	580,000	61.3 (56.7–65.6)	.83
No	630,000	260,000	39.7 (33.4–46.2)	370,000	38.7 (34.4–43.3)	
**Quit intentions^j^**						
Yes	1,040,000	400,000	60.1 (54.5–65.5)	640,000	66.9 (61.9–71.5)	.03
No	580,000	260,000	39.9 (34.5–45.5)	320,000	33.1 (28.5–38.1)	

a Current use of menthol-flavored tobacco products was assessed among students who indicated past 30-day tobacco product use (use of ≥1 tobacco products including e-cigarettes, cigarettes, cigars, smokeless tobacco [chewing tobacco, snuff, dip, snus], dissolvable tobacco products, waterpipes/hookahs, pipe tobacco, bidis, heated tobacco products, and nicotine pouches). Those responding “Yes” to using a flavored product and “menthol” to the type of flavor were categorized as using menthol-flavored tobacco products. For cigarettes, respondents who, within the past 30 days, indicated 1) using only one cigarette brand and indicated that the brand was a menthol-flavored brand (Kool, Newport); 2) responded that they smoked Kool or Newport brands to the question “During the past 30 days, what brand of cigarettes did you usually smoke? (Choose only 1 answer)” (asked among respondents who used multiple brands in the past 30 days); or 3) who answered “Yes” to “During the past 30 days, were the cigarettes that you usually smoked menthol?” were categorized as using menthol-flavored tobacco products.

b Estimated weighted total numbers were rounded to the nearest 10,000 people. Overall population estimates might not sum to corresponding subgroup population estimates because of rounding or inclusion of students who did not self-report sex, race and ethnicity, or grade level.

c *P *value calculated by using the χ^2^ test of independence and indicates whether there are differences between use of menthol-flavored and nonmenthol-flavored tobacco products for each characteristic.

d Unstable estimate is not presented because of a relative SE of ≥0.3 or unweighted denominators less than 50.

e Family affluence was assessed with a composite scale that comprised 4 questions: 1) “Does your family own a vehicle (such as a car, van, or truck)?”; 2) “Do you have your own bedroom?”; 3) “How many computers (including laptops and tablets; not including game consoles and smartphones) does your family own?”; and 4) “During the past 12 months, how many times did you travel on vacation with your family?” Complete data from all 4 questions (n = 1,617 among students who currently used flavored tobacco products) were summed (range = 0–9) and categorized into approximate tertiles based on the sample’s weighted distribution of scores.

f Exposure to tobacco product marketing (advertisements or promotions) was assessed separately for e-cigarettes, cigarettes, and other tobacco products for 4 sources: retail stores; internet; television, streaming services, or movies; and newspapers or magazines. Respondents were asked, “When you [are using the Internet; read newspapers or magazines; go to a convenience store, supermarket, or gas station; watch television or streaming services (such as Netflix, Hulu, or Amazon Prime); or go to the movies], how often do you see ads or promotions for [e-cigarettes; cigarettes or other tobacco products]?” Respondents were categorized as exposed if they responded “sometimes,” “most of the time,” or “always” or unexposed if they responded “never” or “rarely.” Those who reported “I do not use the internet,” “I do not read newspapers or magazines,” “I never go to a convenience stores, supermarket, or gas station,” or “I do not watch television or streaming services or go to the movies” were excluded from the analysis. There were 262 respondents excluded.

g Persons who used tobacco products in the past 30 days who indicated use of any product on 20 or more days in the past 30 days were categorized as using tobacco products frequently; otherwise, if all tobacco products were reported as being used less than 20 days out of the last 30, then persons were categorized as not having frequent tobacco product use.

h Based on the question “During the past 30 days, have you had a strong craving or felt like you really needed to use a tobacco product of any kind?” those answering “yes” were categorized as craving tobacco products within the past 30 days.

i Based on the question, “During the past 12 months, how many times have you stopped using all tobacco products for 1 day or longer because you were trying to quit tobacco products for good?” responses other than “I did not try to quit all tobacco products during the past 12 months” were considered having made 1 or more quit attempts. Respondents (n = 286) missing data on this outcome were excluded from the analysis.

j Based on the question, “Are you seriously thinking about quitting the use of all tobacco products?” Responses of “Yes, during the next 30 days,” “Yes, during the next 6 months,” “Yes, during the next 12 months,” and “Yes, but not during the next 12 months” indicated having quit intentions. The response, “No, I am not thinking about quitting the use of all tobacco products” indicated not having quit intentions. Respondents (n = 265) missing data on this outcome were excluded from the analysis.

**Characteristics associated with menthol-flavored tobacco product use among students who use flavored tobacco products. **We examined correlates of menthol-flavored tobacco product use among middle and high school students who reported current use of any flavored product. Except for sex and intending to quit using all tobacco products, significant associations between covariates and use of menthol-flavored tobacco products remained after adjustment for grade level, sex, and race and ethnicity, although some changes existed in the strengths of association. Compared with non-Hispanic White students, the prevalence of menthol-flavored tobacco product use was lower among Hispanic students (APR, 0.59; 95% CI, 0.45–0.77) and non-Hispanic Black students (APR, 0.34; 95% CI, 0.22–0.53) (Table 3). Compared with students who were not transgender, current prevalence of menthol-flavored tobacco product use was also higher among students who were transgender (APR, 1.45; 95% CI, 1.03–2.03) and those who were not sure if they were transgender (APR, 1.55; 95% CI, 1.14–2.12). Current prevalence of menthol-flavored tobacco product use was also higher among students who indicated frequent tobacco product use (APR: 1.88; 95% CI, 1.59–2.22); use of multiple tobacco products (APR, 1.68; 95% CI, 1.36–2.05); wanting to use a tobacco product within 30 minutes of awakening (APR, 1.55; 95% CI, 1.27–1.88); and craving tobacco products within the past 30 days (APR, 1.34; 95% CI, 1.08–1.66), compared with respective reference categories.

**Table 3:** Univariate and Multivariable Associations Between Selected Characteristics and Use of Any Menthol-Flavored Tobacco Product^a^ Among Middle and High School Students (N = 2,020) Reporting Current Use of Any Flavored Product, National Youth Tobacco Survey, 2022

Characteristic	Unadjusted		Adjusted^b^	
	PR (95% CI)	*P* value	APR (95% CI)	*P* value^c^
**Sex**				
Male	1.22 (1.01–1.47)	.04	1.15 (0.97–1.37)	.11
Female	1 [Reference]		1 [Reference]	
**Race or ethnicity**				
Hispanic	0.61 (0.47–0.78)	<.001	0.59 (0.45–0.77)	<.001
Non-Hispanic Black	0.33 (0.21–0.51)		0.34 (0.22–0.53)	
Non-Hispanic White	1 [Reference]		1 [Reference]	
Non-Hispanic Other	0.93 (0.71–1.21)		0.93 (0.71–1.21)	
**Grade**				
Middle school (grades 6–8)	1 [Reference]	.23	1 [Reference]	.40
High school (grades 9–12)	1.15 (0.91–1.46)		1.10 (0.88–1.38)	
**Sexual orientation**				
Heterosexual	1 [Reference]	.80	1 [Reference]	.45
Gay, lesbian, or bisexual	1.00 (0.79–1.28)		1.00 (0.77–1.31)	
Not sure	1.12 (0.80–1.57)		1.23 (0.91–1.68)	
**Transgender identity**				
No, not transgender	1 [Reference]	.0463	1 [Reference]	.0497
Yes, transgender	1.50 (1.08–2.08)		1.45 (1.03–2.03)	
Not sure	1.57 (1.14–2.16)		1.55 (1.14–2.12)	
I don’t know what this question is asking	1.02 (0.62–1.68)		1.07 (0.61–1.87)	
**Family affluence scale^d^**				
Low	0.96 (0.72–1.27)	.73	0.96 (0.73–1.26)	.64
Medium	0.89 (0.66–1.19)		0.87 (0.64–1.18)	
High	1 [Reference]		1 [Reference]	
**Tobacco product advertising exposure^e^**				
Yes (most of the time/always/sometimes)	1.31 (0.87–1.97)	.18	1.31 (0.89–1.93)	.15
No (rarely/never)	1 [Reference]		1 [Reference]	
**Frequent tobacco product use^f^**				
Yes	2.01 (1.72–2.35)	<.001	1.88 (1.59–2.22)	<.001
No	1 [Reference]		1 [Reference]	
**Use of multiple tobacco products**				
Yes	1.74 (1.39–2.19)	<.001	1.68 (1.36–2.05)	<.001
No	1 [Reference]		1 [Reference]	
**Time to wanting to use a tobacco product <30 minutes after awakening**				
Yes	1.59 (1.31–1.92)	<.001	1.55 (1.27–1.88)	<.001
No	1 [Reference]		1 [Reference]	
**Craving tobacco products within the past 30 days^g^**				
Yes	1.52 (1.23–1.88)	<.001	1.34 (1.08–1.66)	.01
No	1 [Reference]		1 [Reference]	
**Past-year quit attempt^h^**				
Yes	0.98 (0.79–1.21)	.83	0.97 (0.77–1.23)	.81
No	1 [Reference]		1 [Reference]	
**Quit intentions^i^**				
Yes	0.84 (0.71–1.00)	.04	0.86 (0.70–1.06)	.14
No	1 [Reference]		1 [Reference]	

Abbreviations: APR, adjusted prevalence ratio; PR, prevalence ratio.

a Current menthol-flavored tobacco product use was assessed among students who indicated past 30-day tobacco product use (use of ≥1 tobacco products including e-cigarettes, cigarettes, cigars, smokeless tobacco [chewing tobacco, snuff, dip, snus], dissolvable tobacco products, waterpipes/hookahs, pipe tobacco, bidis, heated tobacco products, and nicotine pouches). Those responding “Yes” to using a flavored product and “menthol” to the type of flavor were categorized as using menthol tobacco products. For cigarettes, respondents who, within the past 30 days, indicated 1) using only 1 cigarette brand and indicated that the brand was a menthol-flavored brand (Kool, Newport); 2) responded that they smoked Kool or Newport brands to the question “During the past 30 days, what brand of cigarettes did you usually smoke? (Choose only 1 answer)” (asked among respondents who used multiple brands in the past 30 days), or 3) who answered “Yes” to “During the past 30 days, were the cigarettes that you usually smoked menthol?” were considered as having used menthol-flavored tobacco products.

b Prevalence ratios adjusted for sex, race, and grade level for all variables except sex, race, and grade. APR for sex adjusted for race and grade; APR for race adjusted for sex and grade; APR for grade adjusted for sex and race.

c *P* value was calculated by using the Wald χ^2^ and tests for differences between menthol status groups (menthol flavors, nonmenthol flavor tobacco product use) for each characteristic.

d Family affluence was assessed with a composite scale that comprised 4 questions: 1) “Does your family own a vehicle (such as a car, van, or truck)?”; 2) “Do you have your own bedroom?”; 3) “How many computers (including laptops and tablets, not including game consoles and smartphones) does your family own?”; and 4) “During the past 12 months, how many times did you travel on vacation with your family?” Complete data from all 4 questions (n = 1,617) were summed (range = 0–9) and categorized into approximate tertiles based on the sample’s weighted distribution of scores.

e Exposure to tobacco product marketing (advertisements or promotions) was assessed separately for e-cigarettes and cigarettes or other tobacco products for 4 sources: retail stores; internet; television, streaming services, or movies; and newspapers or magazines. Respondents were asked, “When you [are using the Internet; read newspapers or magazines; go to a convenience store, supermarket, or gas station; watch television or streaming services (such as Netflix, Hulu, or Amazon Prime), or go to the movies], how often do you see ads or promotions for [e-cigarettes; cigarettes or other tobacco products]?” Respondents were categorized as exposed if they responded “sometimes,” “most of the time,” or “always” or unexposed if they responded “never” or “rarely.” Those who reported “I do not use the internet,” “I do not read newspapers or magazines,” “I never go to a convenience stores, supermarkets, or gas stations,” or “I do not watch television or streaming services or go to the movies” were excluded from the analysis. There were 262 respondents excluded.

f Students who used tobacco products within the past 30 days who indicated use of any product on 20 or more days in the past 30 days were categorized as using tobacco products frequently; otherwise, if all tobacco products were reported as being used less than 20 days out of the last 30, then students who used tobacco product within the past 30 days were categorized as not using tobacco products frequently.

g Based on the question “During the past 30 days, have you had a strong craving or felt like you really needed to use a tobacco product of any kind?” Those answering “yes” were categorized as craving tobacco products within the past 30 days.

h Based on the question, “During the past 12 months, how many times have you stopped using all tobacco products for 1 day or longer because you were trying to quit tobacco products for good?” Responses other than “I did not try to quit all tobacco products during the past 12 months” indicated having made 1 or more quit attempts. Respondents (n = 286) missing data on this outcome were excluded from the analysis.

i Based on the question, “Are you seriously thinking about quitting the use of all tobacco products?” Responses of “Yes, during the next 30 days,” “Yes, during the next 6 months,” “Yes, during the next 12 months,” and “Yes, but not during the next 12 months” indicated quit intentions. The response, “No, I am not thinking about quitting the use of all tobacco products” indicated not having quit intentions. Respondents (n = 265) missing data on this outcome were excluded from the analysis.

## Discussion

We found that more than 1 in 5 students who reported current use of at least 1 tobacco product reported use of a menthol-flavored tobacco product. Among students who reported use of at least 1 flavored tobacco product, nearly 2 in 5 reported current use of a menthol-flavored tobacco product. Additionally, 3 in 10 students who reported currently using only flavored e-cigarettes reported using a menthol-flavored product; more than 3 in 10 students who currently only used flavored combustible tobacco products reported using a menthol-flavored product; and more than half of all students who currently used a combination of flavored e-cigarettes, combustible tobacco products, and noncombustible tobacco products reported use of a menthol-flavored product. Differences in sociodemographic characteristics, tobacco product use behavior, and cessation indicators were found among middle and high school students who used menthol-flavored tobacco products.

The prevalence of menthol-flavored tobacco product use was highest among non-Hispanic White students and lowest among non-Hispanic Black students — a result that is contrary to studies focused on menthol cigarette smoking among youths and adults (14,15). At the time of our writing, we found no studies focused on prevalence of any menthol-flavored tobacco product use among youths by race or ethnicity; most studies focused on menthol cigarette smoking or any flavored tobacco product use or did not distinguish between menthol and mint flavors (14,15,17,18). Although our results contrast with some previous studies of cigarette smoking among young people, these findings align with recent research on menthol cigarette smoking that reported a similar pattern (14,19). Miech et al reported that Black adolescents had a lower prevalence of menthol cigarette smoking than adolescents of other races and ethnicities, although results from modeling showed that Black adolescents who smoked cigarettes were more likely to smoke menthol cigarettes compared with White adolescents (19). The results from our study and the Miech study could be partially attributable to a lower prevalence of cigarette smoking in general among young people (12,13) and later-age onset of cigarette smoking among non-Hispanic Black people (20,21). The higher prevalence of e-cigarette use compared with other tobacco products among youths may also play a role. E-cigarettes account for a large proportion of prevalence of any tobacco product use in this population, and fruit- and candy-flavored e-cigarettes are popular in this population (12,13). Populations of young people with a high prevalence of e-cigarette use differ from adult populations with a high prevalence of cigarette smoking relative to other tobacco products. We saw differences by race and ethnicity and among any menthol-flavored tobacco product use (15).

Among students who reported past 30-day use of flavored tobacco products, we saw no association between sexual orientation and menthol-flavored tobacco product use. This is in contrast with previous literature among adults who smoke menthol cigarettes (3). This could be due partly to the high proportion of youths using e-cigarettes and nonmenthol-flavored noncigarette tobacco products (12).

Similar to results from previous studies focused on menthol cigarette smoking (17,22), our study’s results show that, among students who used menthol-flavored tobacco products within the past 30 days, use was associated with behaviors that indicated tobacco dependence. These behaviors include frequent tobacco product use, use of multiple tobacco products, wanting to use tobacco products within 30 minutes of awakening, and craving a tobacco product within the past 30 days. These results suggest use of any menthol-flavored tobacco product (alone or in combination with other flavors) among students who use any flavored tobacco products may be associated with symptoms of dependence, which in turn, can contribute to continued use.

We also found that in 2022, 30.6% of students who currently used only flavored e-cigarettes used menthol e-cigarettes. To our knowledge, our study is one of a few studies focused on the prevalence of menthol-flavored tobacco product use among middle and high school students who currently use any flavored tobacco product, although at least 1 study assessed this among all youths (not just those who currently use tobacco products) (18). Most studies have focused exclusively on the prevalence of menthol cigarette smoking (14,17,19). Thus, our study expands the knowledge base on use by young people of menthol flavor across multiple tobacco product types.

Findings of this study are subject to at least 4 limitations. First, the sample size was not large enough to present characteristics of menthol-flavored product use by exclusive use of individual tobacco product types (eg, cigarette smoking only, cigar use only). Second, NYTS data are cross-sectional, and identified associations reflect tobacco use patterns at the time of survey completion. Third, NYTS data are subject to response bias. However, the validity of self-reported tobacco product use in population-based studies has been shown to be high (23). Finally, our results are generalizable only to middle and high school students in public and private schools in the US.

As of July 2023, menthol is the only nontobacco flavoring allowed in cigarettes sold in the US since the 2009 Family Smoking Prevention and Tobacco Control Act, which prohibited the sale of all characterizing flavors of cigarettes except menthol and tobacco (24). Additionally, in early 2020, the US Food and Drug Administration (FDA) prohibited the use of characterizing flavors in cartridge-based e-cigarettes, excluding menthol (25). In 2022, FDA proposed standards to prohibit menthol as a characterizing flavor in cigarettes and all flavored cigars (6).

Although prohibiting sales of flavors can have a significant effect on reducing tobacco product use among young people, the continued availability of menthol could mitigate the effects of policies prohibiting flavors (26). For example, immediately following the FDA’s announcement of prioritized enforcement of sales of prefilled e-cigarette cartridges in flavors other than tobacco and menthol, increases occurred in the market share of menthol-flavored, prefilled, cartridge-based e-cigarettes and nonmenthol-flavored (including fruit, candy, and alcohol flavored) disposable e-cigarettes (27,28). How this affected overall e-cigarette use among young people is currently unknown. However, a recent study in Minnesota reported changes in tobacco product use in this population after a flavor ban that included menthol was implemented in the Twin Cities (Minneapolis and St. Paul) (26). The study reported that any tobacco product use and e-cigarette use among youths increased to a greater extent in the rest of the state of Minnesota when compared with the increase in the Twin Cities (26). Additionally, use of noncigarette tobacco products with flavors other than mint or menthol by youths increased by 5% in the Twin Cities compared with 10.2% in the rest of the state (26). This shows that the inclusion of menthol in prohibitions of tobacco product flavor can further reduce overall tobacco product use among youths.

As new product types continue to be added to the tobacco landscape, examining the role of menthol and other characterizing flavors or additives in all tobacco products will be important to determine factors that may contribute to initiation and sustained use of tobacco products. Future studies are needed of menthol-flavored tobacco product use with sufficient sample sizes to assess use of specific tobacco products by demographic groups. Continued surveillance of the use of all characterizing flavored tobacco products (including menthol) and the effectiveness of restrictions on flavored tobacco product sales are needed to inform public health policy and tobacco prevention and control efforts.
